# Factors associated with preterm birth at Wachemo University Nigist Eleni Mohammed memorial hospital, southern Ethiopia: case-control study

**DOI:** 10.1186/s12884-020-03503-9

**Published:** 2021-01-07

**Authors:** Negeso Gebeyehu Gejo, Melaku Tesfaye W/mariam, Biruk Assefa Kebede, Ritbano Ahmed Abdo, Abebe Alemu Anshebo, Hassen Mosa Halil, Biruktawit Fekade Woldu, Nuradin Abusha Katiso

**Affiliations:** 1 Department of Midwifery, College of Medicine and Health Sciences, Wachemo University, Hosanna, Ethiopia; 2 Department of Pharmacy, College of Medicine and Health Sciences, Wachemo University, Hosanna, Ethiopia; 3 Department of Public health, College of Medicine and Health Sciences, Wachemo University, Hosanna, Ethiopia

**Keywords:** Associated factors, Preterm birth, Southern Ethiopia

## Abstract

**Background:**

Preterm birth is defined as the birth of a baby before 37 completed weeks of gestation. Worldwide, prematurity is the second foremost cause of death in children under the age of 5 years. Preterm birth also gives rise to short and long term complications. Therefore, the primary aim of this study was to identify the factors associated with preterm birth in Wachemo University Nigist Eleni Mohammed Memorial referral hospital, Hadiya Zone, Southern Ethiopia.

**Methods:**

An institution-based unmatched case-control study was conducted from July 01, 2018 to June 30, 2019 among mothers who gave birth in Wachemo University Nigest Eleni Mohammed Memorial referral hospital. A retrospective one-year data was retrieved from medical records of mothers with their index neonates. Simple random sampling technique was employed to recruit study participants. SPSS version 20 software was used for data entry and computing statistical analysis. Both bivariable and multivariable logistic regression analyses were used to determine the association of each independent variable with the dependent variable. Odds ratio with their 95% confidence intervals was computed to identify the presence and strength of association, and statistical significance was affirmed if *p* < 0.05.

**Result:**

The current study evaluated 213 medical records of mothers with index neonates (71 cases and 142 controls). Urban residency [AOR = 0.48; 95% Cl; 0.239, 0.962], antenatal care follow up [AOR = 0.08; 95 Cl; 0.008, 0.694], premature rupture of membranes [AOR = 3.78; 95% Cl; 1.467, 9.749], pregnancy induced hypertension [AOR = 3.77; 95% Cl; 1.408, 10.147] and multiple pregnancies [AOR = 5.53; 95% Cl; 2.467, 12.412] were the factors associated with preterm birth. More than one-third (36.6%) preterm neonates died in the present study.

**Conclusions:**

The present study found that urban residency, antenatal care follow up, premature rupture of membranes, pregnancy induced hypertension and multiple pregnancies were factors associated with preterm birth. The mortality among preterm neonates is high. Enhancing antenatal care follow up and early detection and treatment of disorders among pregnant women during antenatal care and undertaking every effort to improve outcomes of preterm birth and reduce neonatal mortality associated with prematurity is decisive.

**Supplementary Information:**

The online version contains supplementary material available at 10.1186/s12884-020-03503-9.

## Background

The World Health Organization (WHO) defines premature baby as “babies born alive before 37 weeks of pregnancy are completed.” Each year, a projected 15 million babies are born preterm and this figure is increasing [1]. In the year 2015 alone, an estimated 6 million children under the age of five died globally. Of these, about 2.6 million died within the first month of being born and more than 60% of these deaths occurred in Africa and South Asia. Just over a third of these babies died as a result of complications related with prematurity [2, 3].

In 2014, 10.6% (9.0–12.0) was the estimated worldwide preterm birth rate, making a projected 14.84 million premature babies alive (12.65 million–16.73 million). Of these, 12 million (8.1%) preterm births took place in Asia and sub-Saharan Africa. In the sub-Saharan Africa the estimated preterm birth rate was 12.0 making the proportion of preterm birth 28.2% [4].

Preterm birth gives rise to short and long term adverse outcomes. Adverse outcomes of prematurity are responsible for 35% of worldwide neonatal deaths, and the second top cause of under-5 deaths following pneumonia. The long term severe health consequences include loss of sight, hearing impairment, cerebral palsy, and developmental difficulties, comprising cognitive, sensory, learning and language deficits [3, 5].

Preterm birth imposes substantial expenses to government, and moreover parents frequently face extensive emotional and economical sufferings. Though the risks of death and severe morbidity are much greater in early gestation (< 34 weeks), preterm babies born at late gestation (34–37 weeks) have considerably increased risk of complications than babies born at term [6].

Disparities in survival rates of premature babies are observed across the globe. Half of babies born at 24 weeks stay alive in high-income countries, whereas in low income settings, Half percentage of babies born at 32 weeks continue to die due to absence of feasible and cost-effective care [1].

In 2016, the neonatal mortality rate (NMR) was 28 deaths per 1000 live births in Ethiopia. The neonatal mortality rate varies in rural and urban areas; 43 deaths per 1000 live births and 41 deaths per 1000 live births in rural and urban areas respectively. In Ethiopia, birth asphyxia (31.6%), prematurity (21.8%) and sepsis (18.5%) were the three top causes of neonatal mortality at national level respectively in 2015 [7].

Identifying the risk factors of preterm birth is essential for molding services and initiation of risk specific interventions and preventive measures. Therefore, the aim of this study was to identify factors associated with preterm birth among women who gave birth at Wachemo University Nigist Eleni Mohammed Memorial referral hospital.

## Methods

### Study setting, population and sample size

An institution-based unmatched case-control study was conducted from July 01, 2018 to June 30, 2019 among mothers who gave birth at Wachemo University Nigist Eleni Mohammed Memorial hospital which is located in Hossaena town, Hadiya Zone, South Nation’s Nationalities and Peoples Region at 232kms south of Addis Ababa, capital city of Ethiopia. It provides preventive, curative and rehabilitative clinical services organized in four case teams as outpatient, inpatient, emergency and critical care, maternal, child health and obstetrics and operation theatre. The hospital was selected due to its highest patient and client attendance. It is the largest hospital in Hadiya Zone serving the Zonal population and also neighboring Zones and special woredas such as Silte, Gurage, Halaba and Kembata-Tembaro.

A retrospective one-year data on socio-demographic, reproductive, obstetrics, medical and neonatal characteristics was retrieved from medical records of mothers with their index neonates. The source population incorporated all mothers with their index neonates who gave birth at Wachemo University Nigist Eleni Mohammed Memorial hospital during the study period. The study population encompassed selected mothers with their index neonates who gave birth at Wachemo University Nigist Eleni Mohammed Memorial hospital during the study period.

Cases were mothers with index neonates who gave birth between 28 0/7 weeks and 36 6/7 weeks at Wachemo University Nigist Eleni Mohammed Memorial hospital during the study period, and controls mothers with index neonates who gave birth between 37 0/7 weeks and 41 6/7 weeks at Wachemo University Nigist Eleni Mohammed Memorial hospital during the study period.

The sample size was calculated using open Epi Version 2.3.1 statistical software by considering the following assumptions: proportion of multiple pregnancies among the controls which is 34% and adjusted odds ratio of multiple pregnancy among the controls which is 2.50 [8], 95% Cl, 80% power of the study, control to case ratio of 2:1. Finally, after adding 10% for incomplete medical records, the total sample size was estimated to be 213 (71 cases and 142 controls). The formula used to calculate sample size for the current study is described below:

$$ n=\left(\frac{r+1}{r}\right)\frac{\left(\overline{p}\right)\left(1-\overline{p}\right){\left({Z}_{\beta }+{Z}_{\alpha /2}\right)}^2}{{\left({\mathrm{p}}_1\hbox{-} {p}_2\right)}^2} $$

Where *n* = sample size in the case group

r = ratio of controls to cases

$$ \overline{P} $$ =Average proportion

*Z*_*β*_ =desired power

*Z*_*α*/2_ =desired level of statistical significance

p_1_ =probability of events in control group

*p*_2_ = probability of events in case group

### Sampling technique and eligibility criteria

Medical records of mothers with preterm delivery with index preterm neonates (28 0/7 weeks- 36 6/7 weeks) who met the inclusion criteria were recruited using simple random sampling technique as cases where as medical records mothers with term delivery with index term neonates (37 0/7 weeks- 41 6/7 weeks) following cases and who met the inclusion criteria were selected using simple random sampling technique as controls. Both cases and controls were identified by charts and admission log books. Medical records of mothers with their index neonates who gave birth at Wachemo University Nigest Eleni Mohammed Memorial hospital during the study period were included in to the current study and incomplete medical records of mothers with their index neonates lacking pertinent information like gestational age, obstetrics, medical, reproductive and neonatal characteristics were excluded from the current study.

### Data collection tool and procedure

Data was extracted by reviewing medical records of mothers with their index neonates using a pre-tested and structured checklist. The checklist was developed from different related studies and necessary modifications were carried out [9–11]. The validity and reliability of the instrument was assured using pears correlation and Cronbach’s alpha co-efficient test respectively. Data were collected on socio-demographic data, reproductive characteristics, obstetrics and medical complications, neonatal characteristics. Data was collected by 3 midwives.

### Data quality control

The quality of data was assured by applying properly designed and pre-tested checklist. In addition, training was given to data collectors and supervisor. Data collectors were closely followed by the supervisor and principal investigator daily to ensure completeness of the checklist. The checklist was pre-tested on 5% (4 cases & 8 controls) at Durame general hospital and necessary modifications were carried out by adding variables like non-reassuring fetal heart rate pattern and weight for gestational age and others.

### Data processing and analysis

The collected questionnaire was checked manually for its completeness; and coded and entered in to Epi-data 3.1 and analyzed using SPSS version 20.0. Descriptive statistics was computed. Both bivariable and multivariable logistic regression analyses were used to determine the association of each independent variable with the dependent variable. Initially, variables with *p* < 0.30 at bivariable logistic regression were taken in to multivariable logistic regression model. During multivariable logistic regression backward elimination technique was employed. Odds ratio with their 95% confidence intervals were calculated and statistical significance was affirmed if *p* < 0.05. Hosmer-Lemeshow statistic had a significance of 0.944 indicating that the model is fit. Multi-collinearity was checked for interaction between independent variables through VIF (Variance inflation factor) which showed a value of less than 5.

## Results

### Maternal demographic characteristics

In this study, a total of 213 (100%) medical records of mothers with their index neonates (71 cases and 142 controls) were reviewed. Among cases, median maternal age was 28 years (IQR 26, 36) whereas median maternal age among controls was 28 years (IQR 25, 30). Almost half (49.3%) of cases resided in rural area and 103 (72.5%) controls resided in an urban setting.

### Obstetrics characteristics

Median gestational age among cases was 33.0 weeks (IQR 31.6, 34.1) whereas median gestational age among control was 38 weeks (IQR 36.3, 39.0). Among cases, the median parity was 2 (IQR 1, 4) while median parity among controls was 1 (IQR 1, 3). Five (7.1%) cases had history of miscarriage and four (2.8%) controls had history of miscarriage. Four (5.6%) cases had history of preterm birth while only one control had history of preterm birth. Eight (11.3%) cases had no antenatal care follow-up as only one control had no antenatal care follow-up.

Regarding frequency of antenatal care, 54 (84.7%) cases had four and more antenatal care visits whereas 128 (90.8%) controls had four and more antenatal care visits. Four (5.6%) cases had history of stillbirth while three (2.1%) controls had history of stillbirth. Sixty seven (94.4%) cases had spontaneous onset of labour where as 100 and 37 (96.5%) controls had spontaneous onset of labour. Sixty five (91.5%) and Six (8.5%) cases gave birth through spontaneous vaginal delivery and cesarean section respectively. One hundred and fourteen (80.3%), 20 (14.1%) and eight (5.6%) controls gave birth through cesarean section, spontaneous vaginal delivery and instrumental delivery (Table 1).

**Table 1:** Obstetrics characteristics of mothers who gave birth in Wachemo University Nigest Eleni Mohammed Memorial referral hospital, 2019 (*n* = 213)

Variable	Category	Cases *n* = 71 (%)	Controls *n* = 142 (%)	Total *n* = 213 (%)
Parity	1–2	42 (59.2)	92 (64.8)	134 (62.9)
	3–4	15 (21.1)	29 (20.4)	44 (20.7)
	> = 5	14 (19.7)	21 (14.8)	35 (16.4
History of miscarriage	Yes	5 (7.1%)	4 (2.8)	9 (4.2)
	No	66 (92.9)	138 (97.2)	204 (95.8%)
History of preterm birth	Yes	4 (5.6)	1 (0.7)	5 (2.3)
	No	67 (94.4)	141 (99.3)	198 (97.6)
History of stillbirth	Yes	4 (5.6)	3 (2.1)	6 (3.3)
	No	67 (94.4)	139 (97.8)	195 (96.7)
Antenatal care	Yes	63 (88.7)	141 (99.3)	192 (95.5)
	No	8 (11.3)	1 (0.7)	9 (4.5)
Frequency of antenatal care	1	2 (3.2)	1 (0.7)	3 (1.4)
	2–3	7 (11.1)	12 (8.5)	19 (8.9)
	> 4	54 (85.7)	128 (90.8)	182 (85.4)
Labour	Spontaneous	67 (94.4)	137 (96.5)	204 (95.8)
	Induced	4 (5.6)	5 (3.5)	9 (4.2)
Mode of delivery	Spontaneous vaginal delivery	65 (91.5)	114 (80. 3)	179 (84.1)
	Caesarian section	6 (8.5)	20 (14.1)	26 (12.2)
	Instrumental	–	8 (5.6)	8 (3.8)

### Maternal medical complications

Three (4.2%) cases and only one control had cardiac disease. Four (5.6%) cases and two (1.4%) controls had hypertension. Two (2.8%) and three (2.1%) cases and controls had urinary tract infection respectively. Three (4.2%) cases and two (1.4%) controls had diabetes mellitus. Six (8.5%) and three (2.1%) cases and controls had anemia respectively. None of the cases and controls had human immune deficiency virus (HIV) infection, sexually transmitted infection, pyelonephritis and malaria.

### Obstetrics complications

Two (2.8%) cases and one control had antepartum hemorrhage respectively. Sixteen (22.5%) cases had pregnancy induced hypertension while nine (6.3%) control had pregnancy induced hypertension. More than one-fourth, 20 (28.2%) of cases had premature rupture of membranes and ten (9.0%) controls had premature rupture of membranes. Two (2.8%) cases had polyhydramnios whereas only one control had polyhydramnios. Twenty six (36.6%) cases had multiple pregnancies while 14 (9.9%) controls had multiple pregnancies. Nine cases (12.7) and five (3.5%) controls had non-reassuring fetal heart rate pattern (Table 2).

**Table 2:** Obstetrics complications among mothers who gave birth in Wachemo University Nigest Eleni Mohammed Memorial referral hospital, 2019 (*n* = 213)

Variable	Category	Case *n* = 71 (%)	Controls *n* = 142 (%)	Total *n* = 213 (%)
Antepartum hemorrhage	Yes	2 (2.8)	1 (0.7)	3 (1.4)
	No	69 (97.2)	141 (99.3)	210 (98.6)
Pregnancy induced hypertension	Yes	16 (22.5)	9 (6. 3)	25 (11.7)
	No	55 (77.5)	133 (93.7)	188 (88. 3)
Premature rupture of membranes	Yes	20 (28.2)	10 (9.0)	31 (14.1)
	No	51 (71.8)	132 (91.0)	170 (85.9)
Polyhydramnios	Yes	2 (2.8)	1 (0.7)	3 (1.4)
	No	69 (97.2)	141 (99.3)	210 (98.6)
Multiple pregnancy	Yes	26 (36.6)	14 (9.9)	40 (18.8)
	No	45 (63.4)	128 (90.9)	173 (81.2)
Non-reassuring fetal heart rate pattern	Yes	9 (12.7)	5 (3.5)	14 (6.6)
	No	62 (87. 3)	137 (96.5)	199 (93.4)

### Neonatal characteristics

Among cases; three (4.2%) had extremely low birth weight, 21 (29.6%) had very low birth weight, 43 (60.6%) had low birth weight and four (5.6%) had normal birth weight. Among controls; one had very low birth weight, 30 (21.1%) had low birth weight and 111 (78.2%) controls had normal birth weight. None of the controls had extremely low birth weight (no table).

Almost half of the cases 35 (49.3%) were males and slightly more than half of the controls 76 (53.5%) were males. Among cases; 56 (78.9), 14 (19.4%) and only one were appropriate for gestational age (AGA), small for gestational age (SGA) and large for gestational age (LGA) respectively. Among controls; 133 (93.7%), 6 (4.2%) and 3 (2.1%) were AGA, SGA and LGA respectively. Only one case and one control had congenital anomaly. The type of congenital anomaly was esophageal atresia and club foot; and cleft lip and palate with club foot in the case and control respectively (Table 3).

**Table 3:** Neonatal characteristics in Wachemo University Nigest Eleni Mohammed Memorial referral hospital, 2019 (*n* = 213)

Variable	Category	Cases *n* = 67 (%)	Controls *n* = 134 (%)	Total *n* = 201 (%)
Sex of the neonate	Female	36 (50.7)	66 (46.5)	102 (47.9)
	Male	35 (49.3)	76 (53.5)	104 (52.1)
Weight for gestational age	Appropriate for gestational age	56 (78.9)	133 (93.7)	189 (88.7)
	Small for gestational age	14 (19.4)	6 (4.2)	20 (9.4)
	Large for gestational age	1 (1.4)	3 (2.1)	4 (1.9)
Congenital abnormality	Yes	1 (1.4)	1 (0.7)	2 (0.9)
	No	70 (98.6)	141 (99.3)	211 (99.1)

### Neonatal outcomes

**Fig. 1:**
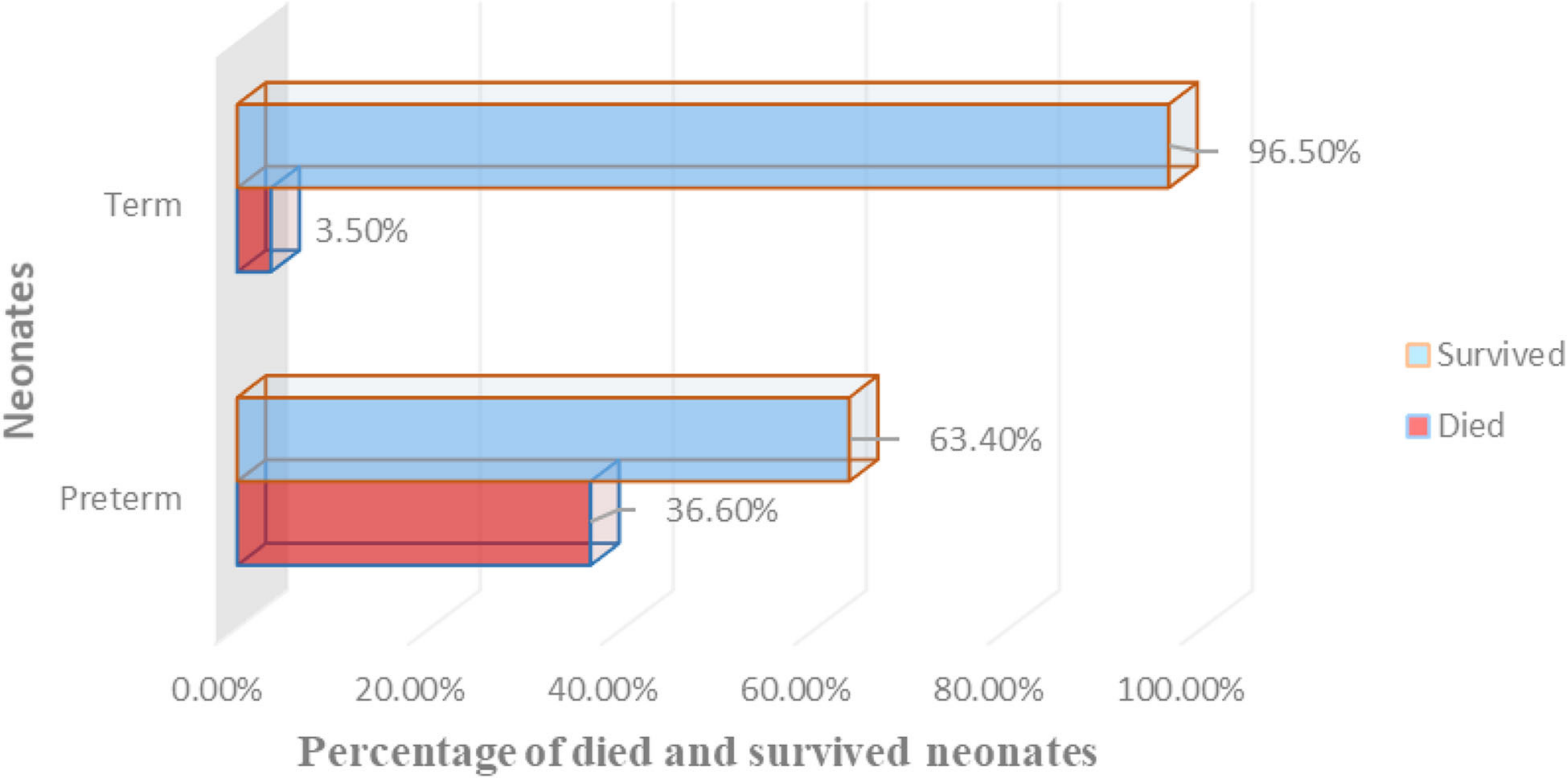
Outcomes of neonates at Wachemo University Nigist Eleni Mohammed Memorial referral hospital, 2019

In the current study, more than one-third of cases died 26 (36.6%) while 45 (63.4%) cases survived. Five (3.5%) and 100 and 37 (96.5%) controls died and survived respectively (Fig. 1).

Among cases, the major possible cause of death, 20 (74.1%) was respiratory failure whereas cardio-respiratory failure 2 (0.4%) and asphyxia 2 (0.4%) were the major possible causes of death among controls (No Fig).

### Factors associated with preterm birth

In bivariable analysis; parity, residency, history of abortion, history of preterm birth, history of stillbirth, urinary tract infection, diabetes mellitus, anemia, ANC follow up, labour, APH, PROM, pregnancy induced hypertension, polyhydramnios and multiple pregnancy were significant at *p*-value < 0.30.

But in the multivariable logistic regression analysis; urban residency, ANC follow up, premature rupture of membranes, pregnancy induced hypertension and multiple pregnancy were significantly associated with preterm birth at p-value less < 0.05 (Table 4).

**Table 4:** Factors associated with preterm birth among mothers who gave birth in Wachemo University Nigest Eleni Mohammed Memorial referral hospital, 2019 (*n* = 213)

Variable	Category	Cases n (%)	Controls n (%)	COR (95% Cl)	AOR (95% Cl)
Parity	1–2	42 (59.2)	92 (64.8)	0.69 (0.318, 1.477)*	0.89 (0.348, 2.252)
	3–4	15 (21.1)	29 (20.4)	0.78 (0.309, 1.946)	0.76 (0.248, 2.342)
	> = 5	14 (19.7)	21 (14.8)	1	1
Residency	Urban	36 (50.7)	103 (72.5)	0.39 (0.215, 0.705)*	0.48 (0.239, 0.962)**
	Rural	35 (49.3)	39 (27.5)	1	1
History of abortion	Yes	5 (7.1%)	4 (2.8)	2.61 (0.680, 10.053)*	2.10 (0.434, 10.168)
	No	66 (92.9)	138 (97.2)	1	1
History of preterm birth	Yes	4 (5.6)	1 (0.7)	8.42 (0.923, 76.775)*	7.93 (0.717, 87.776)
	No	67 (94.4)	141 (99.3)	1	1
History of stillbirth	Yes	4 (5.6)	3 (2.1)	2.77 (0.602, 12.712)*	2.40 (0.365, 15.793)
	No	67 (94.4)	139 (97.8)	1	1
Antenatal care	Yes	63 (88.7)	141 (99.3)	0.06 (0.007, 0.456)*	0.08 (0.008, 0.694)**
	No	8 (11.3)	1 (0.7)	1	1
Labour	Spontaneous	67 (94.4)	137 (96.5)	0.61 (0.159, 2.351)*	2.85 (0.351, 23.190)
	Induced	4 (5.6)	5 (3.5)	1	1
Urinary tract infection	Yes	2 (2.8)	3 (2.1)	1.34 (0.219, 8.225)*	0.41 (0.039, 4.406)
	No	69 (97.2)	139 (97.9)	1	1
Diabetes mellitus	Yes	3 (4.2)	2 (1.4)	3.08 (0.504, 18.918)*	3.89 (0.541, 27.914)
	No	68 (95.8)	140 (98.6)	1	1
Anemia	Yes	6 (8.5)	3 (2.1)	4.28 (1.037, 17.639)*	3. 36 (0.451, 25.07)
	No	65 (91.5)	139 (97.9)	1	1
Pregnancy induced hypertension	Yes	16 (22.5)	9 (6. 3)	4.29 (1.792, 10.313)*	3.77 (1.408, 10.147)**
	No	55 (77.5)	133 (93.7)	1	1
Polyhydramnios	Yes	2 (2.8)	1 (0.7)	4.09 (0. 364, 45.854)	1.14 (0.054, 23.770)
	No	69 (97.2)	141 (99.3)	1	1
Antepartum hemorrhage	Yes	2 (2.8)	1 (0.7)	4.09 (0.364, 45.962)*	1.82 (0.125, 26.435)
	No	69 (97.2)	141 (99.3)	1	1
Premature rupture of membranes	Yes	20 (28.2)	10 (9.0)	5.18 (2.268, 11.812)*	3.78 (1.467, 9.749)**
	No	51 (71.8)	132 (91.0)	1	1
Multiple pregnancies	Yes	26 (36.6)	14 (9.9)	5.28 (2.538, 10.996)*	5.53 (2.467, 12.412)**
	No	45 (63.4)	128 (90.9)	1	1

* = *P*-value ≤ 0.30, ** = *P*-value < 0.05

Mothers who resided in urban areas had 52% reduced odds of developing preterm birth than those mothers’ resided in rural areas [AOR = 0.48; 95% Cl; 0.239, 0.962].

Mothers who had antenatal care follow up had 92% reduced odds of developing preterm birth than those mothers’ who had no antenatal care follow up [AOR = 0.08; 95 Cl; 0.008, 0.694].

Mothers who had premature rupture of membranes had odds 3.78 times higher to experience preterm birth than those who had no premature rupture of membranes [AOR = 3.78; 95% Cl; 1.467, 9.749].

Mothers who had pregnancy induced hypertension had odds 3.77 times higher to experience preterm birth than those who had no premature rupture of membranes [AOR = 3.77; 95% Cl; 1.408, 10.147].

Mothers who had multiple pregnancies had odds 5.53 times higher to develop preterm birth than their counterpart [AOR = 5.53; 95% Cl; 2.467, 12.412].

## Discussion

The presented study was aimed to assess factors associated with preterm birth to confront neonatal morbidity and mortality related with prematurity. After controlling for confounders, urban residency, ANC follow up, premature rupture of membranes, pregnancy induced hypertension and multiple pregnancy were factors significantly associated with preterm birth.

This study found that mothers who resided in urban areas had 52% reduced odds of developing preterm birth than those mothers’ resided in rural areas. This might be due to the fact that women living in urban areas have better access to the health care than in rural area which can play an important part in the prevention of preterm delivery.

Besides, women living in rural areas are more likely to be exposed to hard labour and this increases the risk of preterm delivery particularly to women coupled with other risk factors for preterm delivery. Illiteracy which is more in rural area as opposed to urban area is also an important risk factor for preterm delivery. This finding is supported by other studies [11, 12].

The present study also revealed that mothers who had antenatal care follow up had 92% reduced odds of developing preterm birth than those mothers’ who had no antenatal care follow up. This might be due to the fact that having antenatal care enhance health promotion, detect and prevent complications related with preterm delivery at earliest point. This finding is in line with studies done in central zone of Tigray [8], Debretabour [10] and Jimma [13].

According to the present study, mothers who had premature rupture of membranes had odds 3.78 times higher to experience preterm birth than their counterpart. This might be due to the fact that prolonged premature rupture of membranes will favor microorganisms to ascend to the uterus causing intrauterine infection.

The microorganism will break down the fetal membranes and also produce phospholipase which leads to formation of prostaglandin and endotoxin, substances that stimulate uterine contractions and causing preterm labour. This finding is similar with studies done in Kenya [10], Nigeria [14], Iran [14], Debretabour [10] and Jimma [13].

The current study also verified that mothers who had pregnancy induced hypertension had odds 3.77 times higher to experience preterm birth than those who did not have pregnancy induced hypertension. This might be due to the fact that uteroplacental ischemia in the setting of pregnancy induced hypertension results in adverse pregnancy outcomes including preterm delivery and others. Besides, pregnancy induced hypertension is a frequent reason for terminating pregnancy at early gestation which results in preterm delivery. This finding is in line with studies carried out in Debretabour [10], Jimma [13], Kenya [9], Nigeria [15] and Iran [14].

The other factor associated with preterm birth is multiple pregnancies. Mothers who had multiple pregnancies had odds 5.53 times higher to develop preterm birth than their counterpart. This is due to the fact that multiple pregnancies cause distention of the myometrium leading to uterine contractions and cervical dilation.

Moreover, other obstetric complications like preeclampsia and polyhydramnios concomitantly occur with multiple pregnancies resulting in spontaneous or iatrogenic preterm birth. This finding is consistent with other studies carried out in Tanzania [16], Kenya [9], Central zone of Tigray [8], Jimma [13] and Debretabour [13].

Preterm babies are predisposed to serious illness or death during the neonatal period. Deprived of appropriate treatment, those who survive are at increased risk of lifelong disability and poor quality of life. Complications arising from preterm birth are the main cause of neonatal mortality and the second prominent cause of deaths among children under the age of 5 years [17].

According to the present study, 36.6% % of preterm neonates have died. The possible causes of death were a respiratory failure, apnea of prematurity, necrotizing enterocolitis and perinatal asphyxia. This finding is in line with study done in India in which perinatal mortality was 42.4% and respiratory distress, birth asphyxia and septicemia were common causes of death [18].

The finding of the present study is also consistent with study conducted in Jimma University specialized hospital in which prenatal asphyxia, sepsis, jaundice, low gestational age, respiratory distress syndrome and initial temperature were factors associated with premature infant death [19].

Limitations of the present study include; lack of information on body mass index, antenatal cortical steroids, monthly income and educational status due to retrieving data from secondary source. Larger sample have not been included in the present study due to lack of digitalization in handling of medical records of mothers in the study area (medical records of mothers were handled in a traditional way) and therefore to include larger sample, bigger funds and longer periods are needed. Consequently, smaller sample included in this study have resulted in low and/or absence of some of the chronic medical conditions.

## Conclusions

The present study found that urban residency, ANC follow up, premature rupture of membranes, pregnancy induced hypertension and multiple pregnancies were factors associated with preterm birth. The mortality among preterm neonates is high. The possible causes of death were respiratory failure, apnea of prematurity, necrotizing enterocolitis and perinatal asphyxia.

Enhancing ANC follow up and early detection and treatment of disorders among pregnant women during antenatal care is vital as this will reduce the occurrence of preterm birth. Every effort should be made to improve outcomes of preterm birth and reduce neonatal mortality associated with prematurity. Moreover, qualitative studies should be conducted to explore more factors causing preterm delivery and neonatal mortality associated with it.

## Supplementary Information

**Additional file 1.** Checklist used to assess determinants of preterm birth.

## Data Availability

The datasets used and/ or analyzed for the current study are available from corresponding author on reasonable request.

## Abbreviations

AOR: Adjusted odds ratio
ANC: Antenatal care
AGA: Appropriate for gestational age
COR: Crude odds ratio
LGA: Large for gestational age
NMR: Neonatal mortality rate
PIH: Pregnancy induced hypertension
SGA: Small for gestational age
SPSS: Statistical package for social science
SVD: Spontaneous vaginal delivery
VIF: Variance inflation factor
WHO: World Health Organization
