# Supplementary material for: Factors associated with preterm birth at Wachemo University Nigist Eleni Mohammed memorial hospital, southern Ethiopia: case-control study
Source: BMC Pregnancy Childbirth. 2021 Jan 7;21:35. doi: 10.1186/s12884-020-03503-9 (PMC7792126; doi:10.1186/s12884-020-03503-9)
Supplement: Supplementary file 1 — Additional file 1. Checklist used to assess determinants of preterm birth. [file 12884_2020_3503_MOESM1_ESM.docx]

Annexes

**Checklist used to assess determinants of preterm birth (checklist developed for this study)**

Date ___________________ Questionnaire code________________

**SECTION 1: MATERNAL DEMOGRAPHIC INFORMATION (circle the correct answer/fill in space provided)**

| **S.no** | \| **Demographic Questions** \| \| --- \| | **Responses** | **Skip** | **Code** |
| --- | --- | --- | --- | --- | --- |
|  | Age in years | ________________________ |  |  |
|  | Residency | 1. Urban 2. Rural |  |  |
|  | Parity | _______________________ |  |  |
|  | Gravidity | _______________________ |  |  |
|  | Gestational age of current pregnancy(in weeks) | ________________________ |  |  |

**Section 2: REPRODUCTIVE FACTORS (circle the correct answer/fill in space provided)**

| **S.no** | \| **Obstetrics Questions** \| \| --- \| | **Responses** | **Skip** | **Code** |
| --- | --- | --- | --- | --- | --- |
|  | History of abortion | 1. Yes 2. No |  |  |
|  | Birth Interval | ___________________ |  |  |
|  | History of preterm birth | 1. Yes 2. No | If no go to Q10 |  |
|  | ANC follow up | 1. Yes 2. No |  |  |
|  | If yes how many times | 1. 1 2. 2-3 3. ≥4 |  |  |
|  | Hemoglobin level | 1. <11gm/dl 2. ≥11gm/dl |  |  |
|  | History of stillbirth | 1. Yes 2. No |  |  |
|  | Labour | 1. Spontaneous 2. Induced |  |  |
|  | Mode of delivery | 1. SVD 2. C/S 3. Instrumental |  |  |

**Section 3: Maternal Medical Disorders**

| **S.no** | \| **Obstetrics Questions** \| \| --- \| | **Responses** | **Skip** | **Code** |
| --- | --- | --- | --- | --- | --- |
|  | Cardiac disease | 1. Yes 2. No |  |  |
|  | Hypertension | 1. Yes 2. No |  |  |
|  | HIV infection | 1. Yes 2. No |  |  |
|  | Diabetes mellitus | 1. Yes 2. No |  |  |
|  | Urinary Tract Infection | 1. Yes 2. No |  |  |
|  | Anemia | 1. Yes 2. No |  |  |
|  | Sexually transmitted infection | 1. Yes 2. No |  |  |
|  | Pyelonephritis | 1. Yes 2. No |  |  |
|  | Malaria | 1. Yes 2. No |  |  |

**Section 3: Obstetrics Complications**

| **S.no** | \| **Obstetrics Questions** \| \| --- \| | **Responses** | **Skip** | **Code** |
| --- | --- | --- | --- | --- | --- |
|  | APH | 1. Yes 2. No |  |  |
|  | PIH | 1. Yes 2. No |  |  |
|  | PROM | 1. Yes 2. No |  |  |
|  | Polyhydramnios | 1. Yes 2. No |  |  |
|  | Multiple Pregnancy | 1. Yes 2. No |  |  |
|  | Others | 1. Yes 2. No |  |  |

**Section 3: Neonatal Characteristics**

| **S.no** | \| **Obstetrics Questions** \| \| --- \| | **Responses** | **Skip** | **Code** |
| --- | --- | --- | --- | --- | --- |
|  | Birth weight | _________________________ |  |  |
|  | Sex of the newborn | 1. Female 2. Male |  |  |
|  | Weight for gestational age | 1. AGA 2. SGA 3. LGA |  |  |
|  | NRFHRP | 1. Yes 2. No |  |  |
|  | Congenital abnormality | 1. Yes 2. No |  |  |
|  | Type of congenital anomaly | __________________________ |  |  |
|  | Outcome | 1. Died 2. Survived |  |  |
|  | Cause of death | ____________________________ |  |  |
